# The Effect of Image Count on Accuracy in Digital Measurements in Dentistry

**DOI:** 10.3390/diagnostics14192122

**Published:** 2024-09-25

**Authors:** Neslihan Güntekin, Aslı Çiftçi, Mehmet Gözen, Sema Ateşalp İleri

**Affiliations:** Department of Prosthodontics, Faculty of Dentistry, Necmettin Erbakan University, 42090 Konya, Türkiye; neslihanvarolnv94@gmail.com (N.G.); mehmetgozen@gmail.com (M.G.); semaatslp@gmail.com (S.A.İ.)

**Keywords:** impression, intraoral scanning, trueness

## Abstract

**Objective:** This study investigated how the number of images collected for digital measurements in dentistry affects accuracy compared with traditional methods. **Methods:** A Frasaco maxillary model was scanned using a SHINING 3D AutoScan-DS-MIX dental 3D scanner to create an STL file. The maxilla was molded 10 times using polyvinyl siloxane (Zhermack Elite HD+) to produce plaster models, which were scanned with the same reference scanner to generate 10 STL files. The Frasaco model was scanned 10 times, capturing images in intervals of 800–1000, 1000–1200, and 1200–1500 using a 3Shape TRIOS 3 intraoral scanner, creating additional STL files. These were analyzed with reverse engineering software. **Results:** The most accurate measurements were obtained using 1200–1500 images. Conventional impression techniques performed significantly worse. There was a significant difference between the groups Digital 1200–1500 and Plaster (*p* < 0.001) and between Digital 800–1000 and Plaster (*p* = 0.007). No significant difference was found when the digital groups were compared among themselves. There was also no significant difference between the Plaster and Digital 1000–1200 groups. To compare precision values that were normally distributed across three or more methods, a one-way ANOVA was used. Trueness values that were not normally distributed with three or more methods were compared employing the Kruskal–Wallis test. **Conclusions:** Different image counts affect digital measurement accuracy. The most accurate measurements were obtained when collecting 1200–1500 images. Conventional impression techniques were shown to perform significantly worse than digital impression.

## 1. Introduction

The use of digital technology in dentistry, known as “digital dentistry”, has been one of the most important advances in the field this century. Digital impressions produced with intraoral optical scanners (IOSs) have revolutionized prosthetic treatments and have been instrumental in enabling digital dentistry [1].

With the advent of digital impressions in dentistry, digital and conventional impression techniques have been compared. In contrast to conventional impressions, digital impressions require less workload, shorten measurement time, and provide more precise measurements with good marginal fit. Unlike conventional techniques, digital impressions eliminate the need for laboratory equipment, preventing problems that may occur during the transfer of models and impressions between the laboratory and the clinic. Digital techniques reduce the use of consumable materials, such as plaster and impression materials, and facilitate simple data communication and storage. They are also more convenient and comfortable for patients [2,3,4,5]. However, digital systems require costly equipment and software, which must be kept up-to-date, and technical expertise [2,3,4,5,6].

Acquiring a 3D file, also known as a digital impression or computer-aided impression (CAI), digitizes the area of interest from the patient’s mouth. This is the first step in the digital workflow. Direct digitalization of this process can be achieved using IOSs. As the scanner moves through the mouth, optical sensors and cameras continuously take pictures. The scanner usually captures a series of narrow-field photos or video frame photos or video frames that are combined to create a comprehensive digital model using specialized software [7].

Taking more images during intraoral scanning can improve the quality and accuracy of the 3D model by providing finer details and better coverage of teeth and gums [8,9,10]. However, when the number of images exceeds 1600, the increased data may cause the software to make more errors [11].

Intraoral scanners are subject to technological errors, as they lack fixed references [12]. As such, the initial image produced by the scanner serves as its reference. A best-fit algorithm is used to “stitch” each new image to the preceding one to achieve the greatest possible overlap. Since there is an inherent error in each overlap, the final error tends to progressively increase with each stitching operation. Therefore, errors are expected to be larger with longer scanning fields and more stitching processes [13]. The posterior region has been found to exhibit the greatest discrepancies, measuring up to 170 μm, when the entire arch was scanned [14]. Previous research has compared IOS accuracy [12,13,15].

The conventional method involves taking an impression using type A silicone and producing a plaster model [16]. Alternatively, the impression from a conventional elastomer can be scanned directly or cast into dental stone, and the final 3D file can be obtained using an extraoral or desktop scanner (indirect digitalization). Type A silicone is routinely used in the patient’s mouth for this procedure [17].

The first and most widely utilized digitalization technique since Computer-Aided Design/Computer-Aided Manufacturing (CAD/CAM) technologies were introduced in dentistry was the indirect digitalization of a dental stone model. In fact, most publications still consider indirect digitalization—using a desktop scanner equipped with optical or palpation technology or even a coordinate measurement machine (CMM)—as the gold standard for obtaining a reference model prior to an accuracy comparison [18]. However, the use of IOSs to digitalize the patient’s mouth is becoming increasingly common [17,18].

Digital measurements are expected to be more accurate than traditional methods, as previously shown [19]. However, the use of these techniques remains controversial because measurement accuracy is affected by factors such as the conditions under which the measurement is taken [20]. Studies have shown that the measurement accuracy of IOSs is significantly impacted by scanning time, the number of images obtained per scan, the scanning pattern, and operator skill [21,22], although research on this subject is insufficient. For example, very few articles in the literature have examined the number of images acquired per scan. In this context, we aimed to investigate the effect of the number of images obtained in digital measurements on accuracy and compare it with conventional measurement methods.

The first null hypothesis (H1) of our study is that there will be no difference in overlaying the STL files obtained from the IOS scan with 800–1000, 1000–1200, and 1200–1500 images with the reference STL file. The second null hypothesis (H2) is that IOS measurements will have higher accuracy compared to conventional measurements.

## 2. Materials and Methods

### 2.1. Sample Size Determination

The results of a similar study (*f* = 0.528; α error probability [α err prob] = 0.05; number of groups = 6; number of measurements = 10; correlation between repeated measures = 0.5) were evaluated using the G*Power program (G*Power 3.1.9.6 for Mac; Heinrich-Heine-Universität Düsseldorf, Düsseldorf, Germany), and the required sample size was calculated as 38.41 for a power (1-β error probability) = 0.90 [23]. For four subgroups, *n* = 10 was determined. The precision was calculated as the RMS value among all combinations of superimposed STL files (*n* = 10, C2 = 45). Trueness was evaluated by superimposing the 10 scan datasets onto the reference scan dataset (*n* = 10).

### 2.2. Reference Scanning and Obtaining the STL File

**Figure 1:**
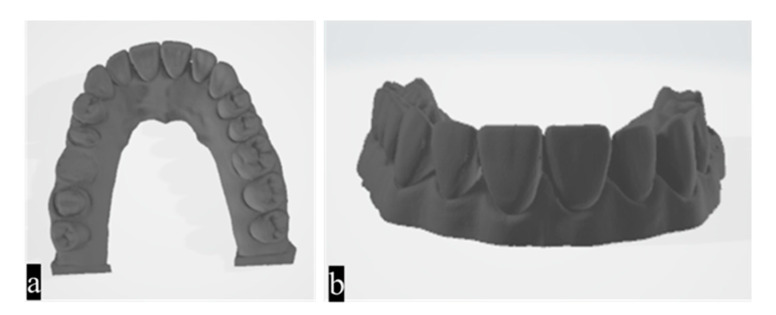
(**a**) Occlusal view and (**b**) buccal view of the reference model STL file.

The Frasaco maxilla model used in the study was scanned using a SHINING 3D AutoScan-DS-MIX dental 3D scanner,(Hangzhou, Zhejiang, China) which served as the reference scanner. Many studies have used dental laboratory scanners to obtain reference data [24]. The AutoScan DS scanner uses blue light technology and has two cameras with a resolution of 5.0 MP and an accuracy of <7 microns. An STL file was obtained as a result. Figure 1 displays the images contained in the STL file corresponding to the reference model [1,25].

### 2.3. Impression Taking and Preparation of the Plaster Model

After scanning the reference model, 10 impressions were taken from the Frasaco maxilla model by the same operator. Polyether (PE) and polyvinyl siloxane (PVS) are the impression materials most commonly used for definitive impressions in fixed prosthodontics. These materials exhibit excellent precision and dimensional stability [26,27]. The impression process was carried out using type A silicone (Zhermack Elite HD+/Zhermack, Italy) in accordance with the manufacturer’s instructions. An appropriate impression tray was selected for the model. The heavy-body material was mixed homogeneously at a 1:1 ratio. The light-body material was mixed homogeneously using a cartridge, and a one-step impression was taken. A waiting period of 3–5 min was observed to allow the impression material to fully set.

**Figure 2:**
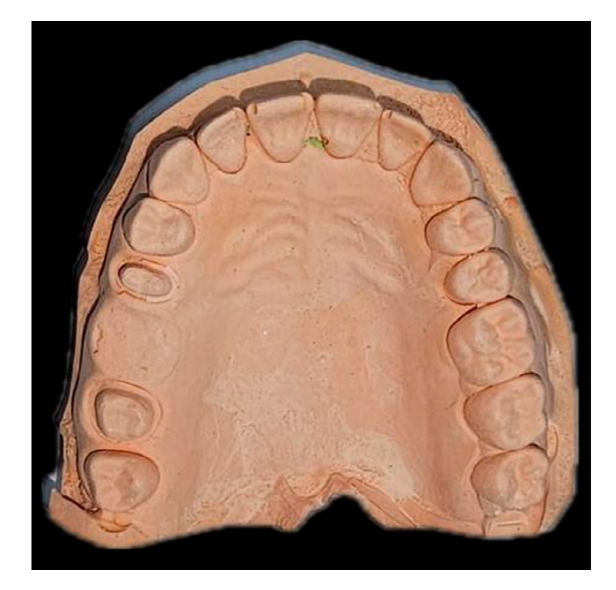
Example of a plaster model.

Immediately after the impressions had set, type 4 plaster was poured into them to create plaster models. We mixed 100 g of plaster powder with 20–25 mL of water, with a setting time of 40 min. The plaster models obtained were scanned using the reference scanner, and an STL file was generated for each model. A total of 10 STL files were created. An example is shown in Figure 2.

### 2.4. Determination of the Minimum Image Count and Photogram Distribution

A total of 20 preliminary scans were conducted to determine the minimum number of images required for a full-arch scan, which was found to be 761. Based on this number, the minimum image count for the operator was set at 800. According to the scanner’s manufacturer instructions, it was recommended not to exceed 1200 images, as going beyond this number increases file size. After completing the full-arch scan, the image count was increased by rescanning. To maintain balanced photogram counts between the groups, a limit of 1500 images was established.

### 2.5. Operator Standardization and Scanning Protocol

All scans were performed by a single operator with over one year of experience with IOSs. Prior to the study, the operator conducted 10 scans of the model to standardize the learning curve associated with the scanner and scanning protocol. During the scanning process, a research assistant monitored the number of photograms and randomly stopped the scan based on the number of images in a given group to ensure the creation of the scanning images.

The operator performed the scans without knowing which group was being scanned; the scan was stopped by the researcher assistant once the required photogram range was reached. While increasing the number of photograms, the same scanning pattern was employed. The assistant researcher regularly evaluated adherence to the protocol and the effectiveness of the blinding procedures.

The scan began in the left posterior region, progressing occlusally toward the right posterior region with a zigzag motion across the anterior teeth. The scan continued buccally toward the opposite side and was completed by moving from left to right on the palatal side. The scanner was calibrated before each scanning session.

### 2.6. Data Analysis and Comparison

During the analysis of the scan results, the groups were created and labeled with letters to minimize the risk of biased decision-making. The accuracy of the obtained STL files was evaluated using CloudCompare v2.13.1. The data were refined by removing unnecessary points (distortions related to undercut areas in the plaster model), and the STL data from the reference model were aligned with those obtained from the IOS. This alignment was performed using the best-fit alignment method, and comparisons were made between the groups.

The data were analyzed using IBM SPSS V23 (IBM Corp., Armonk, NY, USA). The normality of the distribution was checked using the Shapiro–Wilk test. To compare precision values that were normally distributed across three or more methods, a one-way ANOVA was used, and multiple comparisons were examined with Tamahane’s T2 test. To compare trueness values that were not normally distributed across three or more methods, we employed the Kruskal–Wallis test, and multiple comparisons were examined with the Dunn test. The significance level was set at *p* < 0.050.

## 3. Results

The trueness of scan images measured by RMS across different photogram counts is presented in Table 1.

**Figure 3:**
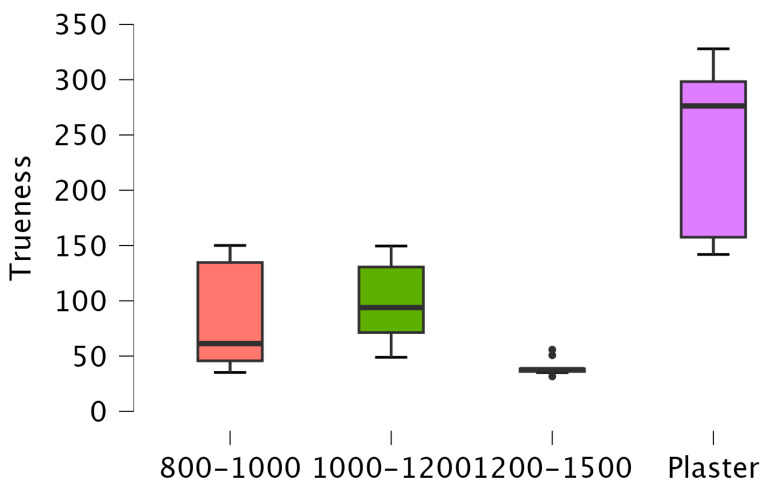
Box plot of RMS trueness values.

There is a significant difference between Digital 1200–1500 and Plaster (*p* < 0.001) and between Digital 800–1000 and Plaster (*p* = 0.007). However, there is no significant difference between Digital 1200–1500 and Digital 800–1000 (*p* = 0.335), Digital 1200–1500 and Digital 1000–1200 (*p* = 0.05), Digital 800–1000 and Digital 1000–1200 (*p* = 1), and Digital 1000–1200 and Plaster (*p* = 0.069) (Figure 3).

The precision of scan images measured by RMS across different photogram counts is shown in Table 2.

**Figure 4:**
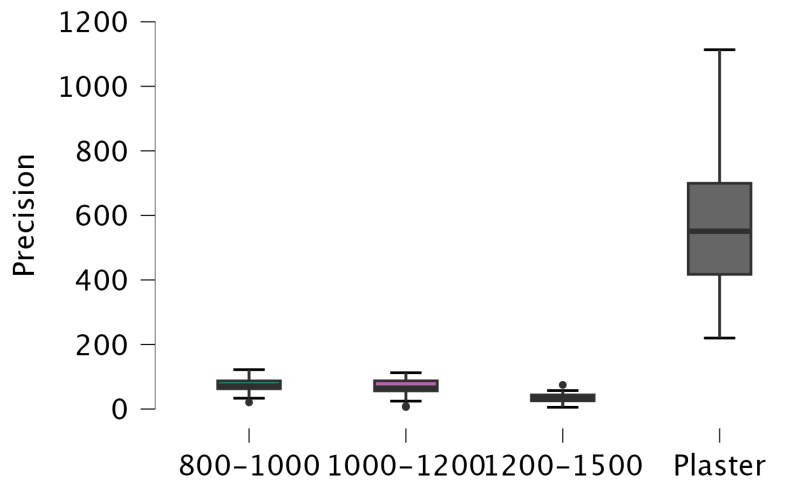
Box plot of precision RMS values.

There is a significant difference between Digital 1200–1500 and Digital 800–1000 (*p* < 0.001), Digital 1200–1500 and Digital 1000–1200 (*p* < 0.001), Digital 1200–1500 and Plaster (*p* < 0.001), Digital 800–1000 and Plaster (*p* < 0.001), and Digital 1000–1200 and Plaster (*p* < 0.001). However, there is no significant difference between Digital 800–1000 and Digital 1000–1200 (*p* = 0.627) (Figure 4).

**Figure 5:**
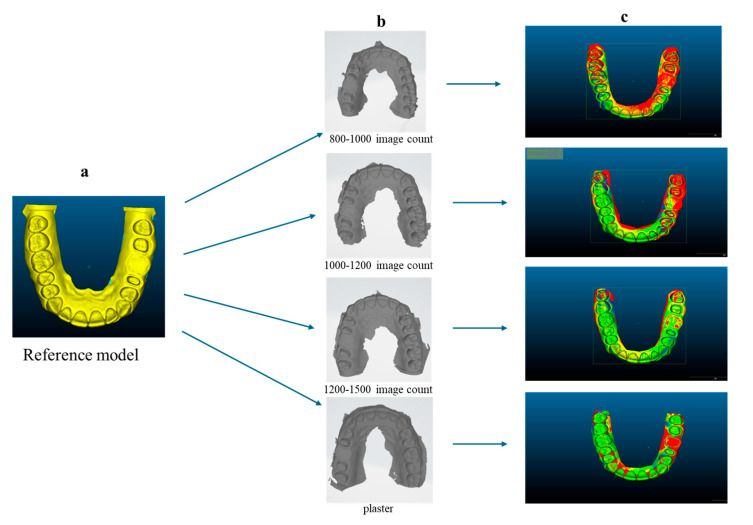
(**a**) STL file of the reference model; (**b**) STL files of the intraoral scans and plaster model; (**c**) amount of deviation after overlapping the scans with the reference model, with C2M signed distances < 0.1; red area = 0.100000 mm; green area = 0.024997 mm; blue area = −0.099995 mm; yellow area = 0.25002 mm.

Figure 5 shows the STL images of the reference model, the digital measurements taken with the IOS, the plaster scan, and the deviation amounts obtained after overlapping all other images with the reference model image.

## 4. Discussion

In this study, our aim was to compare the measurement accuracy of impressions taken with the 3Shape TRIOS 3 (Copenhagen K, Denmark) device for different image counts and the accuracy of conventional impressions with that of digital impressions. It was observed that the number of scanning photograms did not affect the measurement accuracy. Therefore, the first null hypothesis of our study was accepted. However, measurement accuracy was found to differ between the conventional group and the digital groups (except for the 1000–1200 photogram group). Consequently, the second null hypothesis was partially rejected.

In this study, we compared conventional and digital measurement techniques. The digital measurements yielded more successful results than the conventional ones. This is because digital impressions are integrated with CAD/CAM systems, allowing for the design and production of restorations in a digital environment, which results in superior fit and aesthetics. Ng et al. compared the marginal fit of crowns fabricated using digital and conventional methods and found similar results [28]. In addition, if the impression materials are not mixed in the correct ratio and homogeneously during the traditional impression process, the material does not harden properly, thus reducing accuracy. If the plaster is not poured homogeneously and vibrated sufficiently during the modeling process, air bubbles can form in the model, impairing measurement accuracy. Measuring materials are also affected by moisture, temperature, and saliva, which is a disadvantage of traditional measurements [29,30,31].

Numerous factors can impact the accuracy of scanners. For example, the accuracy of intraoral facial cameras is directly affected by the scanning pattern, speed, location, and spatial position of the scanned tooth, oral fluids, anatomical impediments, surface gloss, and the light transmittance of the scanner [32,33,34].

Generally, IOSs capture several images from various perspectives to build a thorough three-dimensional model of the patient’s teeth and gums. This helps in the precise mapping of the intraoral anatomy needed for a variety of dental procedures, including orthodontic treatment, crowns, and bridges [35].

Different scanner models and manufacturers may have different maximum image counts. In our study, the highest measurement accuracy was achieved in the 1200–1500 photograms group, which is consistent with the literature [8,10].

Gomez-Polo et al. analyzed the influence of arch location and scanning pattern on the accuracy, scanning time, and number of photograms of complete-arch implant scans acquired using an IOS (TRIOS 4, Copenhagen K, Denmark) [11]. The authors found that as the number of images increased, trueness also increased, a result similar to ours. In another study, Gomez-Polo et al. concluded that in clinical settings, the duration of the scan or the quantity of photograms obtained may not affect procedures; however, more research is required to assess any potential associations between the number of photograms obtained in intraoral scans and scanning accuracy [23].

Capturing more images per scan can potentially improve the quality and accuracy of the 3D model generated by the IOS. However, whether more images directly translate to better outcomes depends on several factors:

- Resolution and detail: Finer details of the tooth surface and surrounding tissues can be obtained with higher-resolution images that contain more recorded data points. This is particularly crucial for delicate anatomical structures or complex dental restorations.
- Coverage and completeness: Additional photos taken from various viewpoints can ensure thorough coverage of the entire surface of the teeth and gums. This reduces the likelihood that important details will be overlooked during scanning.
- Processing time: Increasing the number of images scanned may also lengthen the time needed for the scanner to compile and process the images into a coherent three-dimensional model. This may impact workflow efficiency, particularly in busy dental clinics.
- Patient comfort: Although IOSs are generally more comfortable for patients than traditional impressions, patient comfort may be influenced by the length of time the scanner remains in the mouth if more images are taken during a scan.
- Scanner technology: The scanner’s underlying technology, such as sensor resolution, scanning speed, and software algorithms, affects how well more images can be captured.

To produce the best results, modern scanners are designed to balance the number of images taken with computational efficiency. In conclusion, while capturing more images per scan may improve the quality of intraoral scans, it is important to factor in processing time, patient comfort, and image quantity and quality. To produce the best results for their patients, dentists often adjust scanning procedures based on specific clinical requirements and the capabilities of their intraoral scanning equipment [8,9,10].

Jamjoom et al. scanned the maxillary and mandibular arches using two IOSs, TRIOS 4 (TRI) and Emerald S (EMR). Polyvinyl siloxane and irreversible hydrocolloids were also used to take conventional impressions of both arches. These impressions were subsequently digitized and compared for accuracy and precision. TRIOS 4 outperformed both conventional methods and the Emerald S scanner (Planmeca, Finland) [36]. Our study yielded similar results

The full-arch accuracy of four distinct IOSs (CEREC Bluecam, CEREC Omnicam, TRIOS Color, and Carestream CS 3500) was assessed by Treesh et al. Carestream CS 3500 exhibited the longest scanning times overall. As expected, it also captured the highest number of images per scan, although its accuracy was lower compared to the other IOSs. This outcome does not align with the findings of our research. The increase in data may result from acquiring more images per scan than the number of photos that the device can match. This may increase the workload on the processor and reduce the scanner’s ability to analyze data quickly and accurately [37].

Sim et al. compared the accuracy of the digital, plaster, and 3D-printed models. In contrast to our findings, the authors found that the 3D-printed model had lower accuracy and that there was no discernible difference in accuracy between the plaster and digital models. Because the impression material used in this study was uniformly thick, the use of individual custom-made impression trays guaranteed the reproducibility of the imprint process. The PVS impression material used in this study exhibited superior dimensional stability compared to other elastomeric impression materials. However, the dimensional stability of the 3D-printed model may be more compromised than that of the plaster model due to shrinkage during the production of layers of minimum thickness with a 3D printer, as well as additional shrinkage and residual stress building during post-curing [38].

To assess the accuracy of crowns made from traditional and digital impressions, Seelbach et al. conducted an in vitro experiment measuring the accessible marginal error and internal concordance. The authors concluded that crowns made with digital impressions were just as accurate as those made with traditional impressions [39].

In an in vivo study, Zarauz et al. assessed the marginal fit of crowns using both digital iTero impressions and traditional silicone impressions. To compare the accuracy of crowns made with digital and conventional impression systems, 26 crowns were made with each method and cemented following standard clinical protocols. The mean internal discrepancy and marginal discrepancy values were measured. The results of the digital system were more accurate, consistent with the results of our study [40].

Similarly, Syrek et al. compared the Lava C.O.S. CAD/CAM crowns’ median marginal space with that of crowns made using the conventional two-stage impression technique. The crowns’ median marginal space was worse when using the traditional impression. Additionally, the interproximal contact points of the CAD/CAM crowns outperformed those of the traditional impressions [41,42]. This study’s findings are consistent with our own. Digital measurements provide high precision, ensuring that the crowns produced are highly compatible with the tooth.

According to the results of our study, the number of photographs taken per scan during digital impression-taking affects precision values. However, there was no significant difference in trueness.

The limitations of our study include the use of a single scanner for creating the reference model and model scans, the lack of assessment of operator experience, the exclusion of different matching algorithms and software, and the fact that it is an in vitro study. Therefore, in vivo studies should be conducted to test different scanners and matching algorithms.

## 5. Conclusions

Different image counts affect digital measurement accuracy. The most accurate measurements were obtained when collecting 1200–1500 images. Conventional impression techniques were shown to perform significantly worse than digital impression.

## Figures and Tables

**Table 1:** Trueness of scan images measured by RMS across different photogram counts.

Trueness								
	Valid	Missing	Mean	Median	Std. Deviation	Minimum	Maximum	F-Value
Plaster ^a^	10	0	237.05	276.3	76.56	141.91	327.93	27.386
800–1000 ^b^	10	0	84.72	61.3	48.96	35.2	150.06	
1000–1200 ^ab^	10	0	98.66	93.9	36.8	48.92	149.56	
1200–1500 ^b^	10	0	39.79	37.7	7.48	31.58	55.82	

Kruskal–Wallis test, ^a, b^: There is no difference between methods that share the same letter.

**Table 2:** Precision of scan images measured by RMS across different photogram counts.

Precision								
	Valid	Missing	Mean	Median	Std. Deviation	Minimum	Maximum	F-Value
Plaster ^c^	45	0	570.89	551	210.44	220	1113.44	134.255
800–1000 ^a^	45	0	74.77	71.4	21.04	20.62	121.98	
1000–1200 ^a^	45	0	67.43	64.7	26.73	6.43	112.56	
1200–1500 ^b^	45	0	35.36	34.6	13.57	5.50	74.73	

One-way ANOVA, ^a–c^: There is no difference between methods that share the same letter.

## Data Availability

The original contributions presented in the study are included in the article, further inquiries can be directed to the corresponding author/s.
